# Breeding of a Wild Population of South Pacific Bonito *Sarda chiliensis chiliensis* (Cuvier 1832) Broodstock under Laboratory Conditions in Pisagua, Northern Chile

**DOI:** 10.3390/ani12010024

**Published:** 2021-12-23

**Authors:** Renzo Pepe-Victoriano, Héctor Aravena-Ambrosetti, Germán E. Merino

**Affiliations:** 1 Facultad de Recursos Naturales y Renovables, Área de Biología Marina y Acuicultura, Universidad Arturo Prat, Avenida Santa María 2998, Arica 1000000, Chile; 2 Programa de Doctorado en Acuicultura Sostenible y Ecosistemas Marinos, Instituto Universitario ECOAQUA, Universidad de Las Palmas de Gran Canaria, Crta. Taliarte s/n, 35214 Telde, Spain; 3 Programa de Magíster en Acuicultura Mención, Cultivo de Recursos Hidrobiológicos y Mención Acuaponia, Facultad de Recursos Naturales y Renovables, Universidad Arturo Prat, Avenida Santa María 2998, Arica 1031597, Chile; hector.aravena.ambrosetti@gmail.com; 4 Sociedad de Servicios Integrales AquaInnova Ltd., Caupolicán 260, Arica 1000000, Chile; 5 Departamento de Acuicultura, Facultad de Ciencias del Mar, Universidad Católica del Norte, Larrondo 1281, Coquimbo 1780000, Chile; gmerino@ucn.cl; 6 Centro de Estudios Avanzados en Zonas Áridas (CEAZA), Unidad Regional de Desarrollo Científico & Tecnológico (CONICYT), Colina El Pino s/n, La Serena 1700000, Chile

**Keywords:** wild-caught broodstock, RAS, spawning, egg incubation, larval culture

## Abstract

**Simple Summary:**

Knowing the biology of fish is fundamental to advance in the culture of wild marine fish species. This is why it is important to form an initial population of broodstock to obtain eggs, larvae, and juveniles of this species for aquaculture research. Therefore, in this research, 24 specimens of “bonito” were captured, transported, and conditioned, and after 14 months in captivity, the fish spawned spontaneously. The eggs were collected and deposited in incubators at 20 °C. By the third day, these eggs had hatched. The newly hatched larvae, as well as the eggs, were characterized during their first morphological changes, which explains that the capture, transport, and conditioning processes were successfully carried out in this research.

**Abstract:**

The wild population of South Pacific bonito *Sarda chiliensis chiliensis*, which has a wide distribution in northern Chile, is considered of importance in Chilean aquaculture. The biological feasibility of cultivation of any marine species begins with the establishment of an initial broodstock population to obtain eggs, larvae, and juveniles. In this work, 22 South Pacific bonito fishing campaigns were carried out in Pisagua, Chile, between spring in November 2011 and the summer in January 2012. At least 74 specimens were obtained of which 24 survived the capture and transport processes. Fish were stocked in a recirculating land-based aquaculture system, and at 14 months under captivity, fish began spawning. Eggs were collected, to describe some stages of development, and were placed in incubators at 20 °C and on the third-day eggs hatched. Larvae reached a total length between 1.435 and 1.7 mm, which were accurately characterized during their first morphological changes. This is the first work that describes the capture, transport, and acclimatization in captivity of a breeding population of wild Pacific bonito in Chile.

## 1. Introduction

The demand for tuna has steadily increased from 0.6 million tones in 1950 to 4 million tones in 2007, leading to the overexploitation of some tuna species, such as yellowfin *Thunnus albacares,* and albacore *Thunnus alalunga* in the Pacific south [1,2,3]. Consequently, the fisheries have focused on relatively underutilized tuna resources, such as skipjack tuna *Katsuwonus pelamis* [4,5,6]. Some small tunas, such as the bonito of the genus *Sarda*, have also been the object of fishing, and among their species, some already show indices of overexploitation, and several researchers are calling for the establishment of fisheries management strategies [7,8,9].

The species *Sarda chiliensis* is a temperate epipelagic schooling fish distributed along the Pacific coast and separated by a tropical zone into two subspecies: the northern subspecies, *Sarda chiliensis lineolata* (Girard, 1858), and the southern subspecies, *Sarda chiliensis chiliensis* (Cuvier, 1832). The northern subspecies is distributed from the coast of Alaska (60°16′ N) to the south to Cabo San Lucas, at the tip of Baja California (22°20′ N) [10]. The geographic range for the southern subspecies ranges from Mancora, Peru (south of the Gulf of Guayaquil) to Talcahuano, in southern Chile.

The management (capture, transport, and conditioning) of broodstock in tanks and the development of a culture technology for this species is a decisive and critical step to improve the sustainable diversification of aquaculture. The present study reviews the technology applied to complete and close the biological cycle of the South Pacific Bonito. These studies and trials have the benefit of improving adaptation to confinement and domestication of a species with a high commercial value for human consumption. In addition, the benefits of the knowledge derived from closing its life cycle imply the improvement of cultivation techniques through the management and control of environmental and biological parameters.

Currently, little biological background, for accurate identification of life cycles [11], and the recognition of embryos and larvae in natural environments [12], types and amount of feed [13,14], reproduction [15], larval development [16], and growth rates [17] is available specifically for the *Sarda chiliensis chiliensis*. Scientific aquaculture studies on the species are rare, which is concerning because of the importance that this resource might have for the diversification of Chilean aquaculture.

South Pacific bonito has a wide distribution in northern Chile and is a candidate species for diversifying Chilean aquaculture. In aquaculture, it is relevant to determine the biological feasibility to rear a new species through the establishment of a first broodstock population to obtain eggs, larvae, and juveniles for research purposes. The two strategies practiced to establish a broodstock are from farmed fish or through the capture of juvenile or adult wild fish [18,19,20,21]. The capture of adult wild fish is complex as several reports indicate injuries caused by fishing gear, handling, and the conditions of transfer to aquaculture facilities. The consequences of an inadequate capture and transfer process will generate uncertainty and instability concerning the viability of the fish as a valuable broodstock. Depending on the degree of stress [22] to which the wild-caught adult fish were subjected, some negative consequences could be expected, such as immunosuppression, primary and secondary pathogenic diseases, gonadal atresia, and decreased egg quality, among others. Due to the sensitivity of wild adult fish, capture strategies recommend using juveniles to initiate the conditioning of future breeders of potentially interesting species for aquaculture [23].

The purpose of this research was to rear a wild-caught population of South Pacific Bonito, *Sarda chiliensis chiliensis* to establish a first breeding stock under laboratory conditions in northern Chile.

## 2. Materials and Methods

**Figure 1:**
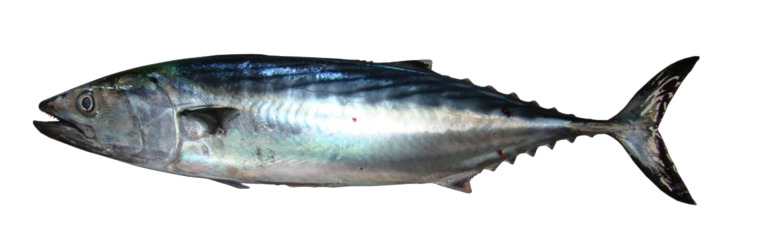
Specimen of South Pacific Bonito (*Sarda chiliensis chiliensis*).

According to previous capture experiences, it has been observed that South Pacific bonito (Figure 1) are very sensitive to manipulation. Therefore, care was taken to maintain minimal contact with the fish skin during all steps involved in the capture and transfer to the land-based RAS facility. The RAS consisted of passing the culture water through a closed circuit, in which it was subjected to filtering and disinfection treatment. This water processing was carried out continuously to eliminate built-up contamination coming from fish waste, food remains, and others. It should be noted that only one experience of ova and larvae culture, corresponding to one spawning, was carried out in the present research for rearing feasibility purposes. For other observed spawnings, the date of spawning, the amount of egg, and the size of eggs were recorded.

### 2.1. Catch and Transportation of South Pacific Bonito

**Figure 2:**
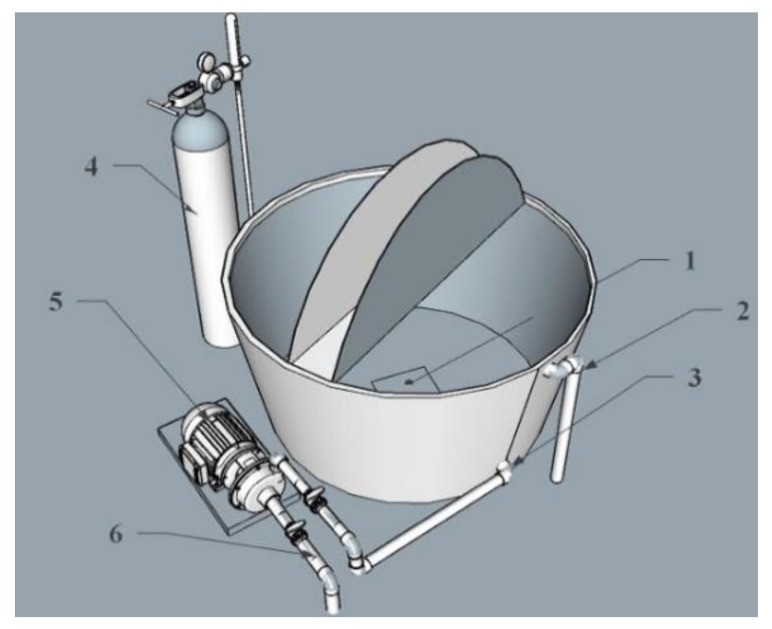
Scheme of the fish transport tank that was installed onboard the fishing vessel. 1. Oxygen outlet; 2. Water outlet from the tank; 3. Water inlet to the tank; 4. oxygen tank; 5. Pump; 6. seawater intake pipe.

A total of 22 fishing campaigns were conducted for South Pacific bonito during the Chilean late spring and summer months, between November 2011 and January 2012. The specimens were caught in the sector of Pisagua in northern Chile (19°36′22.57″ S, 70°12′09.96″ W). A fishing gear called “chispa”, which consists of a fishing line with a barbless hook and a lure, was dragged by a boat at speeds of 2 to 3 knots [24]. Once the fish had taken the hook, it was brought on board and transferred untouched into a 1 m^3^ black fiberglass transport tank. This tank was supplied with continuously flowing oxygenated seawater using a 0.5-hp pump (PedrolloTM) and a 9-m^3^ compressed oxygen gas cylinder that diffused the gas through a ceramic diffuser (Figure 2). Fish larger than 1 kg and smaller than 1 kg were differentiated at the time of their capture, weighing them in a container with water and using a digital balance V-1026 (MoccoTM). For each campaign, 4 or 5 specimens were captured, which were kept in the transport tank on board the vessel at a density that would allow for adequate survival as suggested for *Thunnus albacares* [24]. Sea transport time fluctuated between 1 and 3 h per campaign, depending on the distance from the coast to the capture site and how fast specimens could be captured. The fishes were transferred from the transport tank, located on the vessel board, to another transport tank mounted on a vehicle using trays with black lids to maintain minimal contact with the fish skin. It is worth mentioning that the vehicle-mounted tank had the same design features as the one onboard the vessel. During the fish transport, both the temperature and dissolved oxygen were monitored every one hour using a model YSI 55 oxygen meter. The transport time from land to the culture unit in the La Capilla sector was 5 h.

### 2.2. Land-Based Recirculating Aquacultural System (RAS) for Rearing of South Pacific Bonito

**Figure 3:**
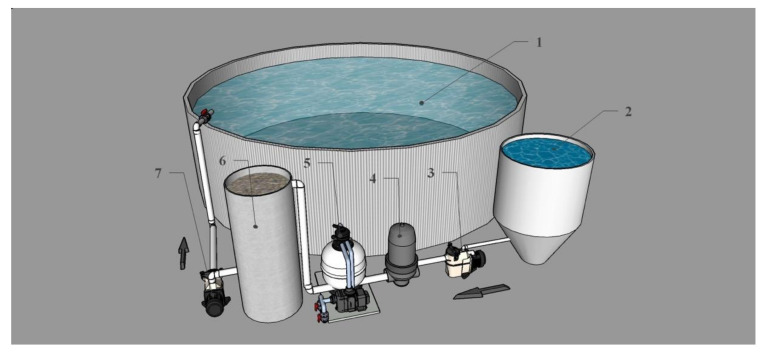
Schematic of the land-based RAS for conditioning South Pacific bonito broodstock: 1 Broodstock rearing tank (75 m^3^); 2 Make-up seawater storage tank (5 m^3^); 3 1.5-hp pump for RAS make-up water; 4 Sand filter; 5 Ring filter; 6 Biofiltration tank (5 m^3^); 7 1.5-hp pump for seawater recirculation.

A land-based recirculating aquaculture system (RAS) was used for rearing a South Pacific bonito broodstock founding population from a captured wild stock (Figure 3). Seawater was continuously recirculated with two 1.5-hp pumps (ReggioTM, model SM150) and treated physically for solids removal and biologically for ammonia removal before re- into the rearing tank. Recirculated seawater was treated for the removal of suspended solids in a linear sequence with a fiberglass settling tank (5000 L), a Hayword sand filter (Blupools, model S360T2), and an Azud ring filter (Azud S.A., modular 100 model). The removal treatment of the culture water of the ammonia excreted by the fishes was carried out using a biofilter (5000 L) containing 3 m^3^ of biomedia (Figure 3). The seawater was aerated within the rearing tank with three diffuser stones (SweetwaterTM, model AS23L) 25 cm long.

A 75-m^3^ cylindrical metallic tank made of steel sheets, corrugated and hot-dip galvanized, and joined with high strength bolts, mounted on a cement floor was used as a rearing tank. The joints were sealed with a flexible asphalt tape that was cured at room temperature. A 1 mm thick plastic liner cover (ASTM D751) was used to contain the seawater in the culture tank. The rearing tank had a diameter of 7.4 m and a water column of 1.76 m. The rearing tank was covered with black mesh to provide 80% shade. The RAS was operated with natural photo and thermo-periods.

### 2.3. Reception and Adaptation to Captive Rearing of South Pacific Bonito

The 75 m^3^ rearing tank was stocked, seven months before South Pacific bonito’s arrival, with four specimens of yellowtail kingfish (YTK) *Seriola lalandi*. The YTKs were obtained from a local farming facility and had an average weight of 800 g. The YTK were used first to provide ammonia for the biofilter start-up and then to facilitate the process of adaptation to the captivity of the South Pacific bonito.

*Sarda chiliensis chiliensis* were continuously observed during the first 24 h after being sown in the breeding tank. In this way, it was possible to record survival after capture from the wild and detect any change in their general external health during their initial adaptation to captive conditions. This procedure was carried out for each of the fishing campaigns. The fish’s records were made visually because it is not advisable to have physical contact with the animals due to their sensitivity. All dead fish were immediately removed, from the rearing tank, during the initial fish adaptation process to the aquaculture facilities.

Fish were fed once a day between 400 to 500 g of a fresh diet consisting of sea silverside *Odontesthes regia* and between 300 and 400 g twice a day of a dry formulated commercial feed for marine fish breeders (Skretting, NOVA ME 2000, and protein percentage 52%). Fresh and dry feeds were offered to South Pacific bonito from Monday to Saturday as recommended for other scombrids [24,25,26,27]. A formalin bath, with a concentration of 1:6000, was carried out in February 2012 for each one of the wild-caught fish to eliminate external parasites, particularly those that could be lodged in the gills.

### 2.4. RAS Water Quality Monitoring

The dissolved oxygen and temperature levels were recorded three times a day using a YSI model 55 oxygen-meter, both in the rearing tank and at the makeup seawater. Ammonium, nitrite, nitrate, and pH were measured in the rearing tank twice a day using a Hanna table spectrophotometer (model HI-83225).

## 3. Results

### 3.1. Catch and Transportation of South Pacific Bonito

A total of 74 South Pacific bonito were caught across 22 campaigns between November 2011 and January 2012 and of which 50 potential broodstock fish did not survive under the conditions used during their tank transport on the vessel. The injuries caused by the catch and the transfer of the selected fish were minimal. Consequently, no treatment with a therapeutic solution was needed to prevent a possible increase in mortalities by management.

The fish survival range for the overall 22 campaigns was between 0% and 100%. There was no correlation between the number of fish transported in the tank (from 1 up to 8 fish/m^3^) and the fish survival. For instance, transport densities of 1 fish/m^3^ resulted in survival rates from 0% up to 100%; 2 fish/m^3^ with survivals between 0% to 60%; 3 fish/m^3^ between 33.3% up to 66.7%; 4 fish/m^3^ between 25% and 75%; 5 fish/m^3^ between 20% and 60%; of 5 fish/m^3^ between 20% and 60%; of 6 fish/m^3^ between 0% and 33.3% (Table 1). A total of 42% of the fish caught were discarded immediately because of the physical damage caused by the fishing gear, mainly considerable injuries to the mouth and gills.

The survivals of the captured fish increased as seasonality transited from late spring to summer. Survival rates for the November, December, and January campaigns were 18.1 ± 14.3%, 29.5 ± 15.3%, and 75.4 ± 17.5%, respectively (Table 1). During the South Pacific bonito fishing campaigns, the seawater temperature ranged between 17.1 and 18.2 °C.

The fish transport density in a vehicle from the dock to the land-based RAS for the cultivation and conditioning of breeding stock did not exceed 4 to 5 fish per tank. The fish transport from the disembarkation dock to the land-based recirculating aquacultural system (La Capilla, Arica) lasted approximately 5 h. There were no mortalities during this transport.

### 3.2. Reception and Adaptation of South Pacific Bonito

Live *Sarda chiliensis chiliensis* that were transferred into the rearing tank were not weighed to minimize handling stress and to cause unnecessary injuries. By the fourth week, it was observed that the first groups of stocked South Pacific bonito began accepting the commercially formulated feed. Fresh and dry daily feed accounted for up to 5.4% of their body weight, assuming an estimated average weight of 1 kg for the 24 stocked fish by the end of the fisheries campaign.

Forty-five days after the last fishing campaign, only two fish (8.3%) died within the broodstock rearing tank. At the end of the 13th month in captivity and one month before the first natural spawning, the accumulated mortality was 11 individuals (45.8%) (Table 2). All 11 dead fish had a compressed abdominal section, and post mortem examination of the fish stomach area showed an empty stomach.

### 3.3. RAS Water Quality Monitoring

**Figure 4:**
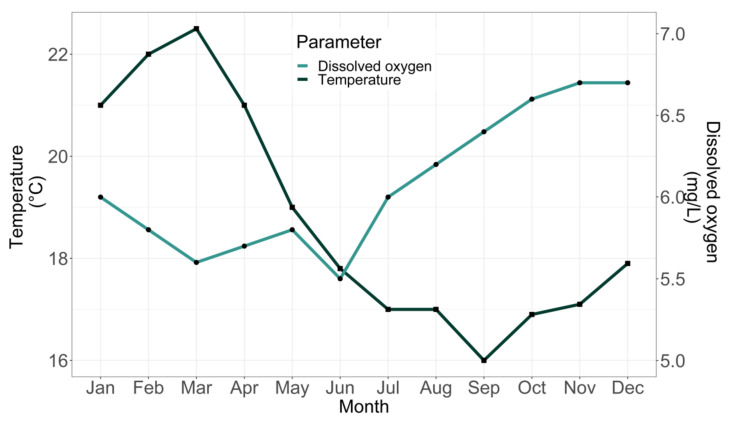
Monthly seawater temperature (°C) and dissolved oxygen concentration (mg L^−1^) in the rearing broodstock tank.

The average temperature and average dissolved oxygen conditions of the water in the rearing tank between January 2012 and December 2012 were 18.80 °C (SD 2.306) and 6.10 mg L^−1^ (SD 0.361) (Figure 4).

The means of ammonium, nitrite, nitrate, and pH in the rearing pond between January 2012 and December 2012 were 0.025 mg L^−1^ (SD 0.996); 0.018 mg L^−1^ (SD 0.888); 35 mg L^−1^ (SD 0.326), and 6.8.

### 3.4. South Pacific Bonito Spawning between January and March 2013

Pacific bonito reached sexual maturity almost 12 months after the last fishing campaign and began to spawn spontaneously in early January 2013 (summer in the southern hemisphere). The spawning lasted until early march of the same year, and at least three weekly spawnings were recorded. The largest number of eggs observed during the spawning period was in February 2013.

According to the history of egg collection, spawning occurred during the course of the morning, which is supported by the fact that the eggs collected, and analyzed between 14 and 16 h, were in states of four blastomeres (110 to 120 min post-fertilization, MPF) to advanced morula (300 to 320 MPF). For all the eggs measured between the neurological and metameric states, it was observed that the vertical diameter of the egg (DHV) with 1.469 ± 0.016 mm was less than the horizontal diameter of the egg (DHH) with 1.622 ± 0.018 mm.

## 4. Discussion

The control of the reproduction of various species of fish has been a definitive step towards achieving production on a commercial scale and the development of fish farming [24,26]. Most of these species were captured to resolve biological feasibility issues for their cultivation in captivity [28]. As one of the most interesting stages of life is that related to the conditioning of potential broodstock animals that can be subjected to the techniques of reproductive control as the first step of domestication, as in the case applied in this research for South Pacific bonito. This study reports a successful attempt to capture and transport wild *Sarda chiliensis chiliensis* to a rearing land-based RAS research facility where they were maintained and eventually spawned naturally.

### 4.1. Capture and Transportation of South Pacific Bonito

The transport of live wild fish is a critical step towards establishing a captive brood fish population, and it is quite difficult to handle fish that have not yet reached their reproductive stage. The acquisition of breeding populations of South Pacific bonito from the natural environment requires special care, which is not usually required for the other development stages, such as the capture of juveniles reported by Flores and Rendíc, [28]. To establish a broodstock population, the native marine fish capture strategy should be designed in such a way that minimal animal handling is required, ensuring the well-being of the specimens caught in their new growing environment [29]. In particular, Scombrids fish must swim constantly since they present negative buoyancy and ram-type ventilation [26]. Capture and transport protocols for the Atlantic bonito *Sarda sarda*, which has been described as a notoriously difficult fish to transport due to its limitations against nitrogen accumulation and dissolved oxygen depletion in the water, have been reported for up to 25 h by road and air [30]. In the case of the South Pacific bonito, its capture was made by fishing them with hooks without barbs to minimize damage to the mouth and gills, as described for *Thunnus albacares* [24], *Euthynnus affinis*, and *Cybiosarda elegans* [26]. This fishing protocol could result in less handling stress and thus positively affect the extension of time observed for acclimatization and conditioning under captive conditions.

Water temperature is an important factor regarding the transport of live fish, and low temperatures are generally suggested to decrease fish metabolism and stress [31]. It was not possible in the 22 fisheries campaigns to control the water temperature in the fish transport tank since the protocol used required a constant flow of fresh seawater. Even though Pacific bonito survival seemed not to be affected by the temperature during their transport. It was observed that overall catches survivability increased as catches progressed from late spring towards summer, with a survival range between 66.7% and 100% for the last catch period on January 2012 compared with catches on November 2011 (average 18.1 ± 14.3%) and December 2011 (average 29.5 ± 15.3%) (Table 1). It was assumed that the increase in the survival of the fish as more catches were made was due to an increasingly better practice of the protocols associated with their handling, managing, and transportation.

Captures of South Pacific bonito in this study between November 2011 and January 2012 show the importance of fish size and transport density in ensuring better survival during transport from the sea to the rearing tank. It was observed that in those catches where the fish exceeded 1 Kg, none of the animals survived during their transport on the boat (Table 1). The lack of survival in fish weighing more than 1 kg, could be attributed to the stress experienced by the fish at the time of capture, as well as the handling and transportation procedures. In contrast, fish smaller than 1 kg were more docile during capture and handling, which resulted in better survival at the end of their transportation and maybe ensured a better adaptation to the captive culture conditions. Similarly, Wexler et al. [24] recommended capturing wild fish smaller than 1 kg and a lower transportation density, which improved survival for *Thunnus albacares*. Bar et al. [26] also reported that *E. affinis* and *C. elegans* weighing less than 1 kg survive the transport, whereas larger fish did not survive. It has been described that physiological disturbances occur in fish during capture, transport, and handling, which reflect some degrees of stress, like (a) primary blood changes (i.e., increased blood levels of ACTH, catecholamine, and cortico-steroids); (b) secondary physiological changes (i.e., changes in oxygen consumption rate, ammonia, and carbon dioxide excretion), and (c) tertiary changes affecting production indexes (i.e., growth rates, survivability) [32]. Meka and McCormick [33] reported that wild specimens of rainbow trout (*Oncorhynchus mykiss*) showed high levels of lactate and cortisol in the plasma, which are recognized indicators of stress after handling and transport. Huax & Sjöbeck [32] found that physiological parameters affected by the capture of wild *Perca fluviatilis* were recovered and stabilized within two to four days after capture. Burke et al. [34] tried unsuccessfully to determine the causes of mortality after 2 or 3 days of capture and delayed mortality up to 20 days in captivity of wild captured *Katsuwonus pelamis*. Davis [35] reported that effects due to capture and handling could lead to reduced growth and delayed mortality and recommended a direct approach of stress conditions was to measure reflex responses after physical stimulation in free-swimming fish. Stressors may be acute (short-term) or also chronic (long-term), and their strength can range from mild to severe, which can be gauged by the induced stress response and its outcomes [36].

Improvements to the catch and transport protocols should be implemented to minimize exposure to stress and increase the survival of wild fish. Suggested methods to reduce stress include: (a) to induce a metabolism reduction by lowering the transport water temperature a few degrees in comparison to the temperature that is registered in the oceanic water for open-water transport broodstock tanks [37]; (b) to apply anesthetics or an injection with a tranquilizing solution [38]; (c) to transport fish under low density or load mass [26]; (d) to design appropriate transport devices for pelagic ram ventilatory fish [26]. In particular, when using wild-caught fish as a broodstock base, it will be necessary to consider all the above recommendations along with the appropriate size of the transport tank, its relationship to the size of the fish, and the water quality requirements during transport [24]. Even though the fish transportation density plays a determining role in fish survivability, that condition itself cannot explain the registered mortalities suffered by *Sarda chiliensis chiliensis* larger than 1 kg. Nevertheless, our transportation density was higher than that reported by Ortega and de la Gándara [39]. During fish transportation, the likelihood of a collision among the fishes could increase significantly, especially when they became disoriented and began to swim against the other fish. Erratic swimming of captured fish had already been reported for *Sarda sarda*, and a way to avoid this problem could be the use of the pipe transport method described by Bar et al. [26].

### 4.2. Reception and Adaptation of South Pacific Bonito

It is not clear in this research whether or not the presence of yellowtail kingfish *Seriola lalandi*, a shoal fish already adapted to the land-based RAS, facilitated the adaptation to the rearing tank and the feeding learning process of the South Pacific bonito. However, it has been already reported that the presence of domesticated shoal fishes provides a better adaptation of wild fish to rearing conditions and an increase in the foraging efficiency, which has been discussed elsewhere [40,41,42,43]. To avoid starvation of wild captured fish and to assure success in their adaptation to the rearing facility, it has been suggested to feed them with fresh dead fish [26] or even live foods [24]. In this research, the Pacific bonitos were fed to an approximate 5.4% of their body mass per day, with a fresh and a dry diet, and it was observed that they began to feed on the commercial diet for marine fish broodstocks (Skretting brand, NOVA ME 2000) approximately four weeks after their introduction to the rearing tank. Yazawa et al. [27] were feeding Eastern little tuna *Euthynnus affinis* with defrosted feed at 5% to 10% of their body weight per day, with fourteen of the 32 initial fish surviving the captivity conditions after one year of rearing in a 70 m^3^, with most mortalities attributed to fish colliding with the tank walls. The daily feed offered to South Pacific bonito in our research was less than the 10% feeding rate offered to condition wild mackerel tuna *Euthynnus affinis* and leaping bonito *Cybiosarda elegans*. Bar et al. [26] reported early mortalities for those fish that refused to feed. In our case, we did not observe initial mortalities when conditioning Pacific bonito, which could imply that our fish were properly fed.

Between November 2011 and February 2012, there were no mortalities in the fish stocked in the rearing tank. However, in the year 2012, after about 45 days of the last fishing campaign, a couple of fish died in march and ten more between June and October (Table 2). The late mortalities recorded allow inferring that all fish that survived transport did not present late mortality and adapted adequately to the culture conditions [12]. The causes of the mortalities were unknown, however, one common feature among the dead fish was the compression of the abdominal section. Right after a fish post mortem examination, it was observed that the stomachs of the fish were empty. The mortality of the breeding stock observed during conditioning was within normal ranges and consistent with that reported for *Graus nigra* [23] and *Dissostichus eleginoides* [44]. The latter authors reported that the highest mortalities for *Dissostichus eleginoides* in the conditioning period occurred in the first rearing months, which was not the case observed here for South Pacific bonito and requires further research to explain it. Bar et al. [26] also reported mortalities of leaping bonito and mackerel tuna after being reared for 6 and 11 months, respectively, and the causes of their mortalities were unknown.

To our knowledge, the mortality of wild conditioned broodstock of *Sarda chiliensis chiliensis* in captivity, observed in our research, would constitute the first report for this novel aquaculture species. In this context, to attribute this reported mortality to the conditioning of bonitos in captivity deserves several hypotheses: first, that although all the fish stocked into the rearing tank were weighing less than one kilogram per fish, as the months of conditioning passed some of these fish made a difference in size which allowed a possible hierarchy of almost 50% of the fish, with favored to more aggressive fish to successfully obtain its feed; second hypothesis that could explain the mortality of the fish would be the that they are visual for fed capture since the water of the rearing tank gradually increased its turbidity, mainly with microalgae, which could have prevented a clear visibility of the fish of the offered feed; a third hypothesis to explain such mortality, is the point of no return in adult fish, perhaps due to the non-feeding of some fish that are the consequence of the hierarchy and/or the water turbidity, which would considerable affect their energy requirements to grow, mature sexually and adapt to captivity; a fourth hypothesis, could be a long term chronic level of stress that the fish could have had in the rearing tank due to endogenous and exogenous conditions, which could present physiological changes caused by confinement, starvation, lighting, and rearing tank size, as reported by Aiyelari et al. [45] for *Clarias gariepinus*. Wang et al. [46] reported a high concentration of cortisol and low concentration of lysozyme for *Perca fluviatilis*, caused by possible adverse conditions present in the rearing tank that produces stress in the fish. It is also worth proposing that post-spawning mortality occurs in several other fish species such as *Perca fluviatilis* [47] and *Psetta maxima* [48], although in our research, spontaneous spawning occurred three months after the last registered mortality.

Ortega and de la Gándara [39] suggested that wild fish taken into captivity must be conditioned as fast as possible to respond early to feed, primarily with fresh food, which will lead the specimens to recover more quickly from the stress of the catch and sharply improve their external appearance. The supply of primarily fresh or frozen diets has generally occurred for the conditioning of wild-caught breeding stock as described by Silva and Oliva [19] for breeding stock of *Paralichthys adspersus* and Muñoz et al. [23] for *Graus nigra*. In our case, fresh diets were provided to South Pacific bonito as a complement to a dry formulated feed, which was appropriate for the species as the fish completed maturation and natural spawning in the rearing tank. The fish showed an adequate adaptation to the feeding regime based on fresh sea bream (*Odontesthes regia*) supplemented with a dry broodstock formulated feed (brand Skretting, NOVA ME 2000). The diet during the conditioning period of the broodstock was most likely adequate as the abundant spawning indicated that the completion of gonadal maturation and the production of good quality eggs.

### 4.3. South Pacific Bonito Spawning between January and March 2013

*Sarda chiliensis lineolata* has been described as a rapidly growing species able to reach 51 cm fork length and up to 1.8 Kg in the first year [17]. Males can mature and spawn at 1 year old, and a few females will spawn at 2 years old, but most will do at 3 years with 69 cm fork length [17]. Most surviving *Sarda chiliensis chiliensis* in this research were less than 1 kg at the time of their stock at the rearing tank and could be within their first year of life. Consequently, most of our fish might have been reaching their second year of life while being conditioned in the rearing tank, which might explain the natural spawning observed at the beginning of the year 2013, as it was reported for *Sarda sarda* [49].

The surviving *Sarda chiliensis chiliensis* grew and matured in the rearing tank, and the latter was evidenced by spontaneous spawning that began in January 2013 in captivity. At the beginning of the spawning period, there were 11 Pacific bonito in the rearing tank, which means a 54% survival rate after one year in captivity, which is similar to the reported survival for *Sarda sarda* [39]. The eggs analyzed in the different spawns released naturally by the *Sarda chiliensis chiliensis* specimens maintained dissimilar characteristics with *Sarda sarda* (e.g., egg diameter, number of oily drops from the egg, and hatching).

It seems that the 75 m^3^ land-based RAS tank size and the general rearing conditions were appropriate for conditioning *Sarda chiliensis chiliensis* as it was for Eastern little tuna *Euthynnus affinis* in a 70 m^3^ land-based open flow rearing tank [27]. However, in our case, the fish had multiple and continuous natural spawning events without the need to administrate GnRHa to induce a spawning as it was required for *E. affinis*.

### 4.4. Present and Future of the South Pacific Bonito in Chilean Aquaculture

Scombids are a pelagic fish family with high aquaculture potential due to their rapid growth and high commercial value. These fish are the subject of major fisheries all over the world. The majority of research efforts have been focused on growing species of the genus *Thunnus*, primarily bluefin tuna. Other species in the family, however, should be considered. Under conditions controlled in captivity, the Pacific bonito, similar to the Atlantic bonito, is a species with a rapid growth reaching up to 1.8 Kg during the first year of life, and males sexually mature at 1 year of life and females at 3 years [17]. The Pacific bonito is a gonochoric species with an asynchronous development of the gonad, and sexes cannot be distinguished using external anatomy [17].

Fishing for wild South Pacific bonito (capture and transport) to establish a suitable broodstock population to develop a rearing technology will constitute the first step to incorporate this novel species towards the diversification and sustainability of Chilean marine aquaculture. This species was able to adapt to the farming conditions in a land-based RAS and started breeding in captivity a year after their capture from the wild. In addition, several spontaneous spawning occurred during the whole summer season [50,51]. These studies and trials have the benefit of improved adaptation to confinement and the domestication of a species with a high commercial value for human consumption and whose life cycle is likely to be fully managed in captivity. Besides, the knowledge benefits derived from closing their life cycle provide the possibility of establishing spawning broodstocks to generate larvae, which will involve the development of cultivation protocols to define methods of species management and requirements for the control of environmental and biological parameters in captivity.

The establishment of this first stock of South Pacific bonito allows other breeders to investigate the biological feasibility of using it as a surrogate species. The South Pacific bonito is a species that is phylogenetically close to the bluefin tuna but with smaller body size and a briefer generation time. The technology to cultivate Pacific bonito should be based on the already widely applied commercial cultures of tuna and marine fish worldwide. In the case of South Pacific bonito, one should expect to be able to adapt the technology used in the cultivation of yellowtail kingfish (*Seriola lalandi*), which is already commercially available near Caldera city in Chile.

## 5. Conclusions

- An initial population of *Sarda chiliensis chiliensis* was established from a wild-caught stock, an important step in improving the diversification and sustainability of Chilean aquaculture.
- All the fish caught and transported were specimens weighing less than one kilogram, which allowed a survival rate of over 63%.
- The wild broodstock was conditioned in a 75 m^3^ rearing tank under a seawater recirculation system, which allowed the first spawning of *Sarda chiliensis chiliensis* in Chile.
- A small population of pelagic fish, *Seriola lalandi*, was used for the process of adaptation to captivity and food learning of *Sarda chiliensis chiliensis*.
- A protocol was established for the capture of potential wild *Sarda chiliensis chiliensis* broodstock and their transport for 5 h to a recirculating aquaculture system on land.

## Figures and Tables

**Table 1:** Weight of wild-caught fish, transport density, and survival of South Pacific bonito in Pisagua.

Capture Date	Number of Fishing Campaign	Total Fish Caught	Ocean Temperature °C	Arrival Temperature °C	Weight of Fish Caught		Fish Density in Vessel Transport Tank Fish/m^3^	Mortality in Capture Tank	Fish Placed into Conditioning Tank		Survival (%)
					Fish > 1 kg	Fish < 1 kg			Fish > 1 kg	Fish < 1 kg	
November	1	6	17.1	21.2	4	2	6	6	0	0	0.0
2011	2	0	17.1	-	-	-	-	-	-	-	-
	3	4	17.3	21.0	2	2	4	3	0	1	25.0
	4	2	17.2	21.2	2	0	2	2	0	0	-
	5	3	17.4	21.3	1	2	3	2	0	1	33.3
	6	4	17.4	21.4	2	2	4	3	0	1	25.0
	7	4	17.6	21.4	2	2	4	3	0	1	25.0
December	8	4	17.5	21.4	2	2	4	3	0	1	25.0
2011	9	5	17.5	21.3	4	1	5	4	0	1	20.0
	10	2	17.6	21.4	0	2	2	1	0	1	50.0
	11	3	17.8	21.3	1	2	3	2	0	1	33.3
	12	6	17.7	21.4	3	3	6	4	0	2	33.3
	13	8	17.7	21.2	5	3	8	6	0	2	25.0
	14	7	17.9	21.3	3	4	7	5	0	2	28.6
	15	1	17.8	21.1	1	0	1	1	0	0	0.0
	16	2	17.8	21.4	0	2	2	1	0	1	50.0
	17	0	17.8								
January	18	3	18.0	21.2	1	2	3	1	0	2	66.7
2012	19	0	17.9	-	-	-	-	-	-	-	-
	20	4	18.2	21.3	1	3	4	1	0	3	75.0
	21	5	18.1	21.2	2	3	5	2	0	3	60.0
	22	1	18.1	21.2	0	1	1	0	0	1	100.0

**Table 2:** Data show the successful campaigns fisheries of South Pacific bonito capture and then stocked in the rearing tank. Cumulative wild captured, stocked fish, fish mortality, and fish population in the rearing tank, during the conditioning and spawning period are shown. Wild-caught South Pacific bonito were fed once daily in the morning with a fresh diet, and twice in the afternoon with a commercial diet. Feeding was offered as soon as the captured fish were stocked in the rearing tank, and continued during the conditioning and spawning period.

		Fish Captured per Month												Conditioning Period											Spawning	
Diet Type	Number of Fisheries Campaign (See Table 1 for Corresponding Dates)	November 2010 Later Spring				December 2010 Summer				January 2011 Summer				February to December 2012											January and February 2013	
		wk1	wk2	wk3	wk4	wk1	wk2	wk3	wk4	wk1	wk2	wk3	wk4	Feb	Mar	Apr	May	Jun	Jul	Aug	Sep	Oct	Nov	Dec	Jan	Feb
Fresh diet once daily																										
Dry diet twice daily	3	1																								
	5		1																							
	6			1																						
	7				1																					
	8 and 9					2																				
	10						1																			
	11 and 12							3																		
	13, 14, and 16								5																	
	17 and 18									2																
	19 and 20										3															
	21											3														
	22												1													
Cumulative wild stocked fish		1	2	3	4	6	7	10	15	17	20	23	24	0	0	0	0	0	0	0	0	0	0	0	0	0
Fish mortality in the rearing tank		0	0	0	0	0	0	0	0	0	0	0	0	0	2	0	0	4	3	1	2	1	0	0	0	0
Fish population in the rearing tank		1	2	3	4	6	7	10	15	17	20	23	24	24	22	22	22	18	15	14	12	11	11	11	11	11

## Data Availability

The data presented in this study are available on request from the corresponding author. The data are not publicly available for privacy reasons.
